# Supplementary material for: Are carbon emissions trading and green financial instruments synergistic? -Comprehensive quantitative research based on content analysis
Source: PLoS One. 2024 Mar 7;19(3):e0298601. doi: 10.1371/journal.pone.0298601 (PMC10919601; doi:10.1371/journal.pone.0298601)
Supplement: S1 Data — (ZIP) [file pone.0298601.s001.zip › Data/地级市Z_F工作报告.zip!/║╙─╧/╞╜╢Ñ╔╜╩╨/└┤╫╘╣1⁄2╓┌║┼í╛┬φ┐╦╩2╛▌═°í┐_2004.docx]

一、过去五年工作回顾

过去五年是我市跨世纪发展的重要时期。五年来，在省委、省政府和市委的领导下，本届政府高举邓小平理论伟大旗帜，认真学习实践“三个代表”重要思想，深入贯彻落实党的十五大、十六大精神，团结带领全市人民，解放思想，抢抓机遇，与时俱进，开拓创新，较好地完成了市七届人大一次会议确定的各项任务，全市各项事业蓬勃发展，改革开放和现代化建设取得巨大成就。

综合经济实力进一步增强。胜利完成了“九五”计划，制定并顺利推进“十五”计划。2003年完成生产总值364．7亿元，五年平均增长8．6％。其中，第一产业增加值增长4．3％；第二产业增长9．9％；第三产业增长8．6％。人均生产总值7480元，年均增长7．9％。地方财政收入14．5亿元，年均增长6．2％；财政支出28．7亿元，年均增长15．7％。金融机构年末各项存款余额314．7亿元，比1998年增加151．8亿元；各项贷款余额210．2亿元，增加53．3亿元。社会消费品零售总额113．5亿元，年均增长9．9％。

经济结构调整成效明显。产业结构进一步优化，三次产业比例调整到12．6∶55．4∶32。粮食总产量144．8万吨，经济作物面积达262万亩，粮经比调整到77∶23。畜牧业产值占农林牧渔业总产值的比重达49．5％，提高8．2个百分点。完成造林绿化102万亩，森林覆盖率达到22％。八大工业基地建设全面推进，五年实施工业结构调整重点项目37个，完成投资77．9亿元，煤炭、电力、冶金、纺织等传统产业得到改造提升，机电、建材、化工、食品等新兴支柱产业得到培育壮大，高新技术产业化及新产品开发工作取得较大进展。大型骨干企业的带动作用增强，工业经济运行质量明显提高，限额以上工业企业综合经济效益指数比1998年提高44个百分点，实现利税27亿元，增长2．1倍，其中利润9亿元，净增11．6亿元。非公有制经济加速发展，实现增加值138．9亿元，占全市生产总值的比重达38％。信息、旅游、房地产、金融保险、现代物流、社区服务等现代服务业呈现良好发展势头。城乡结构调整步伐加快，五年新增城镇人口19．7万人，城镇化水平达到32％。

改革开放取得新的进展。国有大中型企业三年改革脱困目标基本实现，12家大型企业集团建立了比较规范的现代企业制度，平煤、神马、姚电、舞钢4家大型企业通过项目合作、资本引入、债转股等形式，实现了投资主体多元化；长期困扰我市的“三局一矿”改革解困工作取得重大进展。国有中小企业完成改制237户，改制面达91．2％。社会保障体系建设加快。医疗保险走在全省前列；“两个确保”继续巩固，5．6万名国有企业下岗职工基本生活费和5．3万名企业离退休人员基本养老金按时足额发放；建立了城市最低生活保障制度，7．1万名困难居民享受到最低生活保障，实现了应保尽保。再就业工作成效显著，7．4万人实现了再就业，再就业率达69％。各级政府机构改革基本完成，人事制度改革不断深化。市级行政审批事项由1749项减少到358项，市和10个县（市、区）建立了行政审批服务大厅。农村税费改革取得明显成效，农民总体减负38％。财税体制改革取得较大进展，市和10个县（市、区）全部建立了国库支付中心。对外开放提高到新水平，五年新批外商投资企业40家，实际利用外资1．4亿美元，外贸出口年均增长5．6％。日本东芝、日本安川、健力宝集团等一批知名企业相继进驻我市投资创业。

基础设施建设全面加强。集中建设了121个对全市经济具有重要支撑和拉动作用的骨干项目，累计完成固定资产投资283亿元，其中，基本建设投资110亿元。实施国债项目47个，完成投资22亿元。新增公路里程887．2公里，许平南高速公路境内段年底即可竣工，平临、漯平高速公路境内段正加紧施工。五年间，新打机井1．7万眼，增加有效灌溉面积33万亩、旱涝保收田31万亩、节水灌溉面积28万亩，治理水土流失面积495平方公里，农机总动力达到191．5万千瓦。完成了昭平台灌区配套、孤石滩水库除险加固、田岗水库和郏县广阔渠复建工程，白龟山、昭平台、老虎洞水库除险加固等工程顺利实施，燕山水库开工建设。姚电公司1号机组改造、三和热电公司、叶县蓝光电厂一期、城乡电网改造等电力项目建成投产，发电机组总容量达到169．8万千瓦。邮电通信完成投资16亿元，固定电话、移动电话用户分别增长1倍和6倍。加强旅游景区景点开发建设，石人山风景区进入国家4A级景区行列，石漫滩水库和昭平湖景区成为国家水利风景名胜区。

城市面貌明显改观。完成了城市总体规划的修编和新城区控制性详细规划。新建改建29条城市主次干道和11座桥梁，建设路创建迎宾大道、湛河治理、高阳路一期等一批标志性工程建成。对重点地段进行了绿化、亮化，对窗口单位、市民集中活动区及脏、乱、差地段实施了拆迁改造和美化、亮化。新建了鹰城广场、河滨广场、山顶公园和一批街头游园，城市人均公共绿地新增1．27平方米，绿化覆盖率达30．2％。城市公交、供热、供水、排水、燃气等公用事业迅速发展，新建了污水处理厂等一批环卫设施。2003年是城市建设规模最大的一年，老城区改造项目顺利推进，新城区骨干道路和市政大厦等工程正加紧施工，一批与群众生活密切相关的道路和街巷得到建设改造。

人民生活总体上达到小康水平。努力增加城乡居民收入，人民生活总体上实现了由温饱到小康的历史性跨越。五年来，城市居民人均可支配收入年均增长8．9％，农民人均纯收入年均增长2．7％。城乡居民储蓄存款达到231．4亿元，年均增长14．5％。居民食品消费占消费总支出的比重下降，衣食住用行都有较大改善，耐用消费品普及率和档次大幅度提高。住房制度改革稳步推进，出售公房994．3万平方米，新建经济适用房135．6万平方米。“八七”扶贫攻坚计划基本完成，解决了26万农村贫困人口的温饱问题，基本解决了37．6万常年缺水人口的吃水困难。

科技教育和社会事业全面发展。继续实施科教兴平战略，取得科技成果840项，其中省部级88项，国家级2项，10个县（市、区）全部成为国家科技工作先进县（市、区），29家企业、78项产品被认定为高新技术企业和高新技术产品，粉煤灰综合利用位居全国先进行列。坚持优先发展教育，“两基”目标提前实现。高中阶段教育得到加强，在校生数量达到5．6万人；成立了平顶山工学院、平顶山工业职业技术学院和河南质量工程职业学院，平顶山师专升本科工作顺利完成。计划生育综合改革试点工作成绩显著，人口自然增长率下降到4．84‰，连续10年被评为省计划生育工作先进市。合理开发利用水、土地、矿产资源，提高了资源利用效率。加大污染治理力度，生态建设和环境保护工作取得新成效。新闻出版、广播电视和文化事业进一步繁荣，优秀作品不断涌现。医药卫生工作得到加强，在全国率先实行了药品集中招标采购；疾控体系建设步伐加快，夺取了防治非典的阶段性胜利。文化中心、广电中心、急救中心、中心血站投入使用。群众性体育运动蓬勃展开，竞技体育水平有新的提高。军民共建活动进一步深化，连续两届荣获全国“双拥”模范城称号。环保、人防、民族团结和无偿献血等工作被评为全国先进市。统计、审计、外事、侨务、民族宗教、档案、史志、气象、盐业、民兵、对台、技术监督等项工作都取得了新的成绩。

社会主义精神文明和民主法制建设继续加强。“三个代表”学教活动卓有成效。广泛开展“热爱家乡、增辉鹰城”、“道德规范进万家”等群众性精神文明创建活动，广大干部群众的精神风貌和城乡面貌发生了新的变化。拥有国家级文明行业示范点2个、全国文明建设示范单位1个、全国社区建设示范区1个，省级文明行业示范点12个、文明单位182个。坚持依法行政，全面推行政务、村务、厂务公开。严格执行人大及其常委会的决议和决定，自觉接受人大的法律监督和政协的民主监督，办理人大代表议案、建议和政协委员提案2094件，办复率达100％。加强社会治安综合治理，严打整治斗争取得新成果，同“法轮功”等邪教组织进行了坚决斗争。廉政建设和反腐败工作取得新成效。重视人民群众来信来访，不断完善市长热线网络体系，妥善解决群众反映的问题。

过去的五年，在改革与建设的实践中，我们深深地体会到：

发展是解决一切矛盾和问题的根本途径。这五年正值我市改革的攻坚阶段。我们不仅经受了亚洲金融危机、多种自然灾害和非典疫情的冲击，而且面临着国有企业经营困难、结构性矛盾集中凸显、就业压力增大等困难。面对前所未有的严峻形势和困难局面，我们坚持用发展的思路和办法积极应对各种挑战，组织实施了国有企业改革等六大攻坚战，负重奋进，克难攻坚，使经济发展逐步摆脱困境，走上了持续健康的发展轨道。成绩确实来之不易。实践证明，发展是硬道理。把发展作为第一要务，就是要以发展为主题，以发展为大局，只有把握这个大局，突出这个主题，才能调动各种积极因素，破解矛盾，克服困难，实现新的跨越。

改革开放是加快发展的根本动力。作为国有经济高度集中的内陆工业城市，传统的东西根深蒂固，管理体制和运行机制等方面尤其是思想观念与市场经济还不相适应。五年来，我们始终注意把解放思想贯穿到改革开放的各项实际工作中，坚持在干中解放思想，在干中统一认识。尽管思想解放的程度还远远不够，但我们依靠不断解放思想，改革开放的步子不断加快，尤其是国有企业改革，其力度之大、效果之好是多年来所没有的。通过改革开放，调动了各方面的积极性、创造性，为加快发展提供了强大动力。

结构调整是提高经济增长质量和效益的关键。这五年正是我市经济结构转型的重要时期，经济发展中的深层次矛盾，特别是经济结构不合理的矛盾越来越突出，已经成为制约经济发展的重要瓶颈。五年来，我们始终把结构调整作为主线，把工业经济结构调整作为重大战略举措，强力实施工业结构调整重点工程，全面推进八大工业基地建设，不断提高工业经济的竞争力。实践证明，这五年经济发展的历程就是经济结构不断优化的历程。尽管我们面临的经济转型任务十分繁重，但我们依靠不断加大结构调整力度，扭转了经济滑坡的被动局面，实现了速度的增长和质量的提高。

创造良好环境是加快发展的重要条件。五年来，我们始终把环境建设放在突出位置。在优化企业经营环境系列活动中，不断深化行政审批制度改革，强力推进政务公开，建设了行政审批服务中心，强化了源头治理。在整顿和规范市场经济秩序中，坚持突出重点，标本兼治，彻底取缔了小轧钢、地条钢生产和汝州、叶县废旧车辆拆解市场，端掉了叶县马庄乡假烟生产窝点。这五年，是我市在安全生产特别是煤矿安全生产方面勇于探索、大胆创新，实现煤矿安全生产由乱到治的历史性跨越的五年。我们在实践中首创了“三个委派”、严防死守责任制等成功经验，出台了一系列具体规定，先后关停2000余座小煤矿，使安全生产形势逐年稳定好转，得到国家有关部门和省政府的肯定，树立了平顶山的良好形象。

各位代表，过去的五年是我市经济实力明显增强的五年，是改革开放和社会事业全面发展的五年，也是人民群众生活水平不断提高的五年。这些成就的取得，凝聚着全市人民的辛劳和智慧，是方方面面关心支持、共同奋斗的结果。在此，我代表市人民政府，向全市的工人、农民、知识分子和各级干部，向人民解放军驻平部队、武警官兵和公安干警，向驻平单位、各民主党派、工商联、各人民团体、离退休老同志，向所有关心、支持我市现代化建设的各界人士，表示崇高的敬意和衷心的感谢！

在充分肯定成绩的同时，我们也清醒地看到，当前我市经济社会发展中还存在一些突出矛盾和问题。生产总值、工业增加值、财政收入等主要经济指标增速和固定资产投资处于全省中下游水平，与提前5年全面建成小康社会要求差距很大；经济发展的结构性矛盾依然突出，能源原材料工业比重大，高新技术产业发展不快，非公有制经济在国民经济中的比重偏低；城乡二元结构性矛盾突出，县域经济发展不平衡且总体落后，农民增收困难，小城镇尤其是建制镇建设水平偏低，加快城镇化进程任务艰巨；困难企业下岗职工较多，就业再就业压力大；经济外向度低，对外贸易和招商引资成效不理想。同时，一些地方和部门中官僚主义、形式主义、奢侈浪费等现象依然存在，政府机关干部工作作风有待于进一步改进。高度重视并切实解决这些问题，是新一届政府的重要任务。

二、今后五年政府工作的指导思想和目标任务

今后五年政府工作的指导思想是：高举邓小平理论和“三个代表”重要思想伟大旗帜，认真落实党的十六大和十六届三中全会精神，树立全面、协调、可持续的发展观，以全面建设小康社会统揽全局，以“建设大城市，发展大工业”为总体目标，实施二次创业，打造经济强市，大力推进工业化、城镇化和农业现代化进程，不断壮大县域经济和全市综合经济实力，提高整体竞争力和可持续发展能力，促进国民经济持续快速协调健康发展和社会全面进步，努力建设经济更加繁荣、社会更加进步、环境更加优美、人民生活更加殷实的现代化大城市，为率先全面建成小康社会奠定坚实基础。

经过五年的奋斗，努力实现以下几个方面的突破：

经济发展要实现新跨越。积极调整经济结构，切实转变经济增长方式，实现由单一型经济结构向多元化经济结构的转变，封闭型经济向开放型经济的转变，粗放型经济增长方式向集约型经济增长方式的转变，国有经济占绝对比重向多种经济成分共同发展的转变。在保持较高经济增长速度的基础上，实现较高的增长质量。全市生产总值年均增长11％以上，到2008年突破650亿元，财政收入不低于生产总值增长速度；二、三产业增加值占生产总值的比重提高到90％以上；城镇化率达到40％以上。

改革开放要开创新局面。加快经济体制和运行机制创新，以产权制度改革为主线，大力发展股份制经济。继续推进国有企业改革，建立和完善国有资产监管运营体系。农村改革进一步深化，各项配套改革同步推进。毫不动摇地鼓励、支持和引导非公有制经济发展，2008年非公有制经济占全市生产总值的比重达到50％以上。加大招商引资力度，全面提高对外开放水平。发挥高新技术产业开发区和西部新区的优势，做大做强开放型经济园区。

特色经济要形成新优势。优化整合工业资源，拉长产业链条，促进生产要素向大型骨干企业集中，建设在全省、全国有重要影响的煤炭生产和综合加工基地、化工化纤基地、坑口火力发电基地、特宽特厚钢板基地、输变电成套设备和环保设备生产基地、水泥建材工业基地、井盐生产加工基地、原酒生产出口和农副产品加工基地，加快形成独具特色的产业优势，提升区域竞争实力。

城市建设要展现新形象。全面推进城市现代化、农村城镇化、城乡一体化进程，构建双组团、带状式城市形态。强化功能分区，逐步在老城区形成商贸娱乐区，在东部形成工业区，在西部形成科教行政区和生态旅游区。抓好新城区重大项目建设，初步形成六大功能区的总体建设格局，确保三到五年内地下设施基本配套，地面道路纵横成网，城市景观初具规模，建成区面积达到10平方公里，人口达到10万左右，使之成为展现平顶山风采的精品城区。加快城镇建设，构建系统功能完善的网络式城市格局。

小康生活要迈入新阶段。到2008年，全市人均生产总值达到12000元以上，超过全省平均水平。城镇居民人均可支配收入年均增长8％，农民人均纯收入年均增长7％左右。城乡居民人均住房面积分别增加7和5平方米，恩格尔系数下降4个百分点。社会保障体系比较健全，社会就业相对充分，全民思想道德素质、科学文化素质和健康素质明显提高。

今后五年，是实施二次创业、打造经济强市的关键五年。本届政府要始终把发展作为第一要务，按照“五个统筹”的要求，坚持以人为本，认真把握好以下几个事关全局的重大问题：

第一，要把发展工业作为立市之本。牢固树立“工业立市、工业富市、工业强市”的指导思想，确立工业经济在国民经济中的核心地位，确立工业化在推进城镇化和农业现代化中的主导地位。按照走新型工业化道路的要求，集中发展三大产业集群，培育八大工业基地，把工业真正做大做强，增创特色经济新优势。

第二，要把振兴县域经济作为强市之基。按照工业首位、民营主体、项目带动、城乡一体的总体思路，统筹城乡经济发展，以工业的理念发展农业，强力推进县域工业化，带动城镇化和农业现代化，从根本上解决“三农”问题，实现各县（市）综合经济实力在全省的位次明显前移。

第三，要把推进城镇化作为重要战略举措。以纳入中原城市群为契机，强力推进城市建设的跨越式发展，基本实现由矿业城市向功能完善的区域性中心城市转变，构建起主城区支撑有力、县城区功能互补、小城镇竞相发展的现代化大城市的发展框架，依靠城市的率先发展和集聚效应辐射带动全市经济社会发展。

第四，强力推进项目带动战略。紧紧抓住东部发达地区产业向中西部地区转移尤其是国家对煤电资源进行整合的机遇，集中力量建设一批对区域经济发展起决定作用的重大项目，靠项目整合资源，靠项目优化结构，靠项目加快发展，靠项目增强后劲，全面提升区域竞争实力和可持续发展能力。

第五，继续扩大对内对外开放。坚持以改革促开放，以开放促发展，把对外开放作为强市富民和全面建设小康社会的主战略，推进全方位、多层次、宽领域的对外开放，保持我市在国家开放开发和中原崛起战略格局中的竞争优势。

第六，进一步优化发展环境。加强服务政府、法治政府和责任政府建设，提高公务员素质，全面推进政府职能转变；深化企业经营环境整治活动，整顿和规范市场经济秩序，加大源头治理力度，把市场经济秩序逐步纳入法制化管理轨道，为“实施二次创业，打造经济强市”营造宽松环境。

三、2004年的政府工作

2004年是实现“十五”计划的关键一年，也是新一届政府任期的第一年。全市经济和社会发展的主要目标是：全市生产总值增长10．5％；全社会固定资产投资增长18％；社会消费品零售总额增长11％；外贸出口增长12％，直接利用外资增长25％；地方财政收入增长10％；城镇居民人均可支配收入和农民人均纯收入分别增长8％和6％；人口自然增长率控制在5．8‰以内；新增城镇就业人口5万人，城镇登记失业率低于4％。为完成上述目标任务，要认真做好以下十项工作：

（一）加大工业结构调整力度，走新型工业化道路

全面推进经济转型和产业升级。按照走新型工业化道路的要求，推进生产要素向支柱产业集中，向大型骨干企业集中，培育一批主业突出、核心竞争能力强的大型企业和企业集团，集中发展三大产业集群：一是采用高新技术和先进适用技术，改造提升煤炭、电力、冶金、建材等传统支柱产业，整合资源，优化存量，保持其在同行业的领先地位；二是把机电、化工、纺织、食品等优势产业作为接续替代产业的主攻方向，通过改造培育和产业聚集，拉长产品链条，发展高端产品，营造规模优势，使其尽快成为新的支柱产业；三是大力发展装备制造、环保、制药、新型材料、电子信息等高新技术产业，通过优先开发、重点扶持，加速产业化步伐，培育后发优势，使之逐步成为对全市经济具有带动作用的新兴主导产业。

加大结构调整力度。围绕培育八大工业基地，发挥煤、盐、铁、铝等资源优势，大力发展煤电转化、岩盐和煤炭深加工项目，重点发展精制盐、盐化工、煤化工、精细化工和冶金、建材等产业。继续抓好一批结构调整重点工程，开工建设中盐皓龙50万吨工业盐、神马集团5万吨烧碱和5万吨树脂以及新增10万吨尼龙66盐、天瑞集团日产5000吨干法水泥生产线等项目；争取开工建设舞钢公司100万吨中厚钢板生产线。加快天瑞集团铁路重载高速重要部件配件和庇山矿、梨园矿务局宁庄矿扩建等项目建设进度。积极利用再生资源，大力发展循环经济，推动原材料输出向精深加工转变，由初级产品向终端产品生产转变。

加快企业技术创新步伐。鼓励有条件的企业争创省级和国家级技术中心，不断增强自主技术创新能力。加大对重点行业、骨干企业和重点产品的技改力度，突出抓好29项重点技改项目，做好神马集团工业丝扩产改造、天鹰集团高压隔离开关生产线、汇源公司年产15万吨氧化铝等项目的投产达产工作。大力实施名牌战略，深入开展“质量兴市”活动，充分发挥政策性资金的导向作用，积极争取省工业结构调整专项资金、高新技术产业化资金和中小企业创业基金，对省市级名牌产品进行持续扶持，尽快形成一批市场占有率高、生产规模大的名牌产品。

进一步深化国有企业改革。加快组建市国有资产管理机构，建立健全国有资产监督管理体系。按照建立现代企业制度的要求，规范和完善公司法人治理结构，加快分流安置富余人员，分离企业办社会职能。以建立现代产权制度为重点，对国有企业进行规范的股份制改造，加快投资主体多元化的改革步伐，大力发展混合所有制经济。今年要全面完成中小企业的改制工作。稳步推进公用事业企业改制，燃气、自来水、公交等企业要逐步建立起新的运营机制。加强市场体系建设，更好地发挥市场机制对资源配置的基础性作用。

（二）高度重视“三农”问题，推进农业现代化

把解决好农业、农村、农民问题作为各项工作的重中之重，并且毫不动摇地长期坚持下去。继续推进农业和农村经济结构的战略性调整，大力发展优质小麦、专用玉米、“双低”油菜等优质粮油生产，积极抓好优质蔬菜、烟叶、瓜果等高效经济作物生产，畜牧业发展要以扩大规模养殖为重点，加快发展猪、牛、羊、禽四大产业，大力发展畜产品加工业，实现多层次加工转化增值。林果业发展要继续以生态工程造林为重点，抓好山区造林、退耕还林和平原林网建设。大力发展农产品加工业、劳务输出等非农产业

大力推进农业产业化经营。发挥比较优势，发展特色经济。按照“扶大、扶强、扶优”的原则，重点扶持带动农户多、科技含量高的骨干龙头企业。完善公司＋农户办法，强化龙头企业的带动作用。加强农业质量标准体系、农产品检验检测和认证体系、农产品市场体系建设，今年要基本完成市与县（市、区）信息网络建设，建立农产品信息发布和市场准入制度。重点扶持汝州“三粉”批发市场、叶县马庄畜产品批发市场、宝丰粮食批发市场和鲁山张良、汝州纸坊蔬菜批发市场等有一定规模的农产品市场，帮助和引导农民发展绿色农业、精准农业、设施农业、创汇农业和品牌农业。

落实党的农村政策，深化农村改革。认真落实中央一号文件，全面促进农民增收。切实落实农业税率降低3个百分点的政策，落实对种粮农户的直接补贴。稳定以家庭承包为基础、统分结合的双层经营体制，按照依法、自愿、有偿的原则，积极推进土地承包经营权流转机制改革。实行最严格的耕地保护制度，严禁乱占乱用农村耕地。深化粮食流通体制改革，加强粮食市场管理和宏观调控，维护正常的市场秩序和价格体系。继续深化农村税费改革，加强农民负担监督管理，防止农民负担反弹。

加强农业基础设施建设。加快农村道路、水电、通信网络建设，加强农业资源综合开发，提高农业机械化水平。搞好农田水利基本建设，加快燕山水库、昭平台灌区节水续建、孤石滩灌区节水改造和节水增收示范项目建设，恢复建设田岗水库灌区渠系配套工程。组织好农村“六小工程”建设。

（三）加快发展第三产业，培育新的经济增长点

把发展服务业作为重要经济增长点，提高第三产业在国民经济中的比重。发展现代商贸业和物流业，引导流通企业整合资源，推行连锁经营、物流配送和电子商务，加快培育现代物流主体，实现产业升级。深化住房制度改革，建立住房补贴和廉租住房制度，发展住房二级市场和租赁市场，搞好普通商品房和经济适用房建设。鼓励居民扩大旅游、通信、教育、体育和文化消费，积极发展信息、金融、保险、会计、审计、咨询、法律服务、科技服务等中介服务业，拓展新的消费领域。

高度重视旅游业发展，努力把旅游业尽快培育成为我市的支柱产业。加大旅游市场开拓、开放力度，大力推进旅游资源资本化运作、旅游项目市场化开发，通过独资、合资、合作、出让经营权等形式，吸引各类投资主体参与景点推介、开发和基础设施建设。整合旅游资源，开发旅游精品，拉长旅游产业链条。争取实现石人山景区创建国家级文明景区目标；做好石漫滩、风穴寺、三苏园等景区创建A级旅游景区工作，并力争进入国家2A景区序列，培育各具特色的旅游品牌体系。启动创建中国优秀旅游城市工作，舞钢市创建工作要取得阶段性成果。

（四）加快重点项目建设，加大对外开放力度，大力发展非公有制经济

坚持政府导向与企业行为相结合，完善重大项目的策划、实施和储备机制，突出抓好工业、交通、能源、基础设施等81个重点项目。昭平台水库除险加固、县城电网改造、舞钢公司技改、银龙集团扩产等项目年内要建成投产或投入使用；天广水泥公司扩建、天瑞集团张村矿、白龟山水库除险加固、平煤集团郏县大型焦化、升达纸业、宝丰氧化铝等项目要加快建设进度。加快西气东输天然气利用工程建设，新建改造供水、供气、供热管网52公里。抓住国家对全国煤电资源进行整合的机遇，加快平东热电2×20万千瓦热电机组、平煤集团九矿坑口电厂二期、天瑞集团2×13．5万千瓦热电联产机组改扩建的建设进度；开工建设姚电公司2×60万千瓦机组；做好平东电厂二厂2×90万千瓦机组、华电汝州4×90万千瓦机组等项目的前期工作。积极创造条件，加快永平煤电公司汝州矿区能源重化工集群项目的前期工作，力争年内部分项目开工建设。继续大力加强交通建设。加快许平南高速公路境内段工程建设进度，确保年内建成通车；完成平临和漯平高速公路路基和桥涵工程；尽早启动太澳高速公路境内段工程的前期工作。新修改建国省干线公路473．6公里；新修县道二级路80公里；完成296个平原村通油路工程；着力实施好15个山区乡166个行政村道路“千里攻坚”任务，改善山区交通状况。

实施开放带动战略，努力扩大利用外资规模。采取项目融资、股权投资、经营权转让、企业并购、境外上市等方式，吸引更多的境外公司和境外机构来我市投资。加快制造业、服务业、建筑业和基础设施利用外资步伐，拓宽教育、文化、卫生等社会事业利用外资渠道。精心组织好各类招商引资活动，采取网上招商、代理招商等现代招商方式，提高招商引资效率。加强对外商投资项目的协调服务，重点抓好已签约项目的跟踪落实，提高利用外资质量。优化出口商品结构，保持和发展帘子布、钢板、焦炭等传统出口商品份额，努力扩大高压电器、电收尘器等机电产品和农产品出口。扩大对外经济技术合作和劳务输出，鼓励和支持有比较优势的企业到国外投资办厂，开展对外直接投资。

大力发展非公有制经济。要坚持放胆、放手、放开的原则，认真落实鼓励非公有制经济发展的政策措施。进一步放宽市场准入，大力支持非公有资本进入法律法规未禁入的一切行业领域。进一步消除政策歧视，改进服务和监督，确保非公有制经济在投融资、税收、土地使用和对外贸易等方面，与其他企业享受同等待遇。拓宽非公有制企业的融资渠道，引导商业银行加大对非公有制企业的信贷服务。重点扶持比较优势明显、带动作用强的龙头企业，引导非公有制企业走规模型、科技型、外向型发展道路。

（五）大力推进城市发展，加快城镇化步伐

进一步加强城市规划工作。尽快完成城市总体规划的编制、报批，着手编制城市分区域控制性详细规划。做好城市道路交通体系规划，以“八纵六横”14条道路的改扩建规划为重点，完善道路网络；继续做好中心城区景观、绿地和“都市村庄”、城郊接合部的规划建设。进一步加强城市规划管理，加大规划监察执法力度，严格实施规划公示制度、审批制度、业务会议制度、批后管理制度。

加强城市基础设施建设，提高中心城市辐射能力。按照“优化东区功能、改善老区环境、发展西部新区”的发展思路，搞好城市重点工程建设。今年要以高水平建设市政大厦和公务员小区为龙头，带动新城区规划区的基础设施建设。加快主干道、排污管网、市政配套管线建设，搞好办公设施、居民住宅、学校、医院、商业网点等工程建设，为各类机关、事业单位和城乡居民尽快入驻新城区创造条件。要进一步加快老城区改造进度，提升城市品位，努力为居民提供良好的生活环境。搞好城市出入口建设，有计划地打通市区“断头路”和“丁字路”，形成较为完善的道路交通网络，加快程平路建设，搞好开发一路、鹰城广场西路等一批道路和湛河三座大桥工程建设。实施景观工程、亮化工程，强力推进拆墙透绿工程，建设市区小游园22个、主干道人行天桥或地下通道5座，改造市区支路巷道14条。加强环卫设施建设。搞好水、电、气、热、通信、绿化、美化等改造、配套工程。开工建设市污水处理厂二期工程。加快联盟路和文化宫成片改造、干休所区域联片改造及和平路步行街改造工程建设进度。搞好东入市口绿化美化和西部生态园建设。要把高新技术开发区作为对外开放、招商引资的重要窗口，在加快发展上给予倾斜和支持。

加大城市经营管理力度。树立经营城市理念，盘活城市土地和城市资产，多渠道筹措建设资金。进一步规范城市建设投资公司和土地储备中心运作方式，提高运行效益。继续开展土地收购储备工作，规范土地市场，实现土地收益最大化。全面推行拍卖城市道路、桥梁、公园、广场等市政设施的冠名权、广告经营权的作法，开辟城建资金来源。深化投融资体制改革，构建城市建设项目融资平台。积极吸纳社会资本参与城市建设。继续开展市区城市环境综合整治活动，积极探索市政环卫、园林绿化管理改革的新路子，将垃圾收集、运输、处理和保洁、园林管护及城市广场、公园维护工作逐步推向社会，形成城市长效管理机制。

加快推进城镇建设。以县城为重点，加快城镇规划的编制和基础设施、公用设施建设，大力推进城镇基础设施的资产经营，吸纳社会资金投入城镇建设。在省定五个重点镇的基础上，选择一批特色突出、基础较好、潜力较大的建制镇，争取入围国家级重点镇，争得国家的政策和资金支持。引导乡镇企业、各类专业市场向县城和小城镇集中，形成产业支撑，带动和繁荣周边农村经济。进一步消除城镇化的体制性障碍，深化户籍制度改革，做好城镇迁入人口的各类社会服务，促进进城农民与城市的融合。

（六）大力开展增收节支，切实做好财税金融工作

建立适应市场经济要求的公共财政体系，坚持依法治税，依率计征，加强征管，做到应收尽收。深化财政体制改革，完善县乡财政体制。全面推行国库集中收付和政府采购制度，细化部门预算。认真落实“收支两条线”规定，做到收支完全脱钩、收缴彻底分离；加强财政投资评审，强化审计监督，提高财政资金使用效益。调整支出结构，确保党政事业单位工资发放和正常运转，继续增加再就业资金投入和社会保障支出，加大对农业、农村教育和科技的投入。树立过紧日子的思想，严格控制和压缩一般性支出。

加大金融对经济的支持力度。增加各金融机构在我市的贷款投放，扩大对结构调整、基础设施建设、农业生产的贷款支持，确保国债项目配套贷款及时到位；建立和完善贷款担保机制，帮助中小企业和个体私营企业解决贷款难问题。落实国家助学贷款和下岗职工小额担保贷款政策，支持贫困生就学和下岗职工再就业。改善农村金融服务，推广小额信贷和联保贷款，加大对农业产业化龙头企业的信贷支持。完善金融监管机制，坚决惩处逃废银行债务行为，创建金融安全区。继续大力清理整顿“三会一部”。

（七）坚持预防为主，强化安全生产管理

安全生产事关全市改革、发展和稳定的大局，事关人民群众的根本利益。要从讲政治的高度，全面落实安全生产责任制，大力推行质量标准化，强化对安全生产的执法监督，加大安全事故责任追究力度，切实做好安全生产管理工作。要把煤矿安全生产作为安全生产工作的重中之重，坚决落实市政府关于安全生产工作的五个政府令和严防死守责任制，进一步加大稽查力度，严厉打击违法违规生产行为，绝不允许非法无证煤矿继续存在，绝不允许不符合安全生产条件的矿井继续生产，绝不允许违法蛮干行为继续发生。

要高度重视非煤矿山和消防、道路交通、水上交通、建筑、食品卫生和大型特种设备、游乐设施的安全管理，切实加强对烟花爆竹等易燃易爆物品的生产、销售、储运等各环节的管理，突出对公共聚集场所和重点部位的防火安全检查，加强对事故隐患的排查和整顿，加强对重大危险源的监控，防止重特大伤亡事故的发生。

（八）做好就业再就业和社会保障工作，认真解决关系人民群众切身利益的问题

把就业再就业纳入政府目标管理，千方百计开辟就业再就业渠道。加大再就业资金投入，确保税费减免、小额贷款、社会保障补贴等扶持政策落到实处。大力发展劳动密集型产业、服务业，帮助下岗失业人员在社区物业、托幼养老、家政服务等领域实现再就业。大力发展职业培训，继续实行就业援助。切实做好高校毕业生的就业指导和复员转业军人的安置工作。加大劳动保障监察力度，切实维护劳动者合法权益。

继续做好“两个确保”和城市“低保”工作，搞好“三条保障线”的衔接。继续扩大城镇养老、医疗、失业、工伤等社会保险覆盖面，强化社会保险费的征缴、清欠和基金的监督管理。积极发展社会保障事业，做好社会福利、社会救济、优抚安置和社会互助等工作。逐步完善农村特困户生活救助制度，确保困难群众有饭吃、有衣穿、有房住、有病能得到及时医治，不让学生因家庭贫困而失学。

加大农村扶贫开发力度，努力实现2．1万贫困人口脱贫；着手建设农村安全饮水工程，逐步解决缺水、水源不洁地区群众的吃水困难；切实解决建设领域拖欠务工人员工资问题；保证农村中小学教师工资按国家现行标准发放；重视特定传染病的预防救治工作，落实国家对患者实行免费治疗的政策；认真解决征地、拆迁中的补偿问题，依法做好失地农民和城镇拆迁户的补偿、安置工作；加强药品、食品安全卫生检查，实施药品、食品放心工程。

（九）实施“科教兴平”和人才强市战略，繁荣各项社会事业

大力推进科技进步与创新。继续深化科技体制改革，加快完善以企业为主体的技术创新体系和以科研院所为主体的知识创新体系。加强与大专院校和科研院所的合作，加快科技成果转化步伐。加大科技投入，全面推行课题制和科研项目招投标制，引导社会资金投向高新技术的研究开发，促进高新技术产业化。继续搞好农村科普工作，组织实施科技兴县示范推进工程。

大力实施人才强市战略。坚持优先发展教育，全面深化素质教育。深化农村教育改革，加快农村教育发展步伐。巩固“普九”和扫盲成果，提高“两基”水平。加大教育投入，进一步改善办学条件。大力发展学前教育，普及高中阶段教育。扩大高等教育规模，搞好平顶山工学院和平顶山学院建设。加快推进基础教育、职业教育和成人教育“三教统筹”。鼓励社会力量办学，规范教育秩序，提高办学质量。积极推进事业单位人事制度改革，探索建立适应现代企业制度要求的选人用人机制，加大农村人才资源开发力度，为各类经济组织提供人才服务，创造吸引人才、留住人才的环境，促进人才合理流动。

实施可持续发展战略。推进计划生育工作综合改革，加快计划生育法制化进程，落实人口与计划生育目标管理责任制，稳定低生育水平，保持我市计划生育工作在全省的先进位次。依法加强环境保护和生态建设，以防治大气污染为重点，全面推进“蓝天工程”；规范建设项目环境管理，严禁新上污染项目；突出水污染防治，加大白龟山水库综合整治力度。深化国土资源有偿使用制度改革，大力开展矿产资源管理秩序治理整顿，搞好数字平顶山地理空间信息基础设施建设，提高资源的开发利用率。

全面推进文化建设。实施文化精品战略，繁荣文学艺术、广播电视和新闻出版事业。加快“三馆一站”等公益性文化设施建设，做好文物古迹的保护和开发工作。依法加强文化市场管理，加大文化娱乐场所整顿力度。加快广电网络数字化建设，年内初步建成50套数字电视节目平台。深化医疗卫生体制改革，大力发展社区卫生服务，建立健全突发公共卫生事件应急处理体系、疾病防控体系和执法监督体系。抓好市第一人民医院病房楼、平煤总医院综合楼和市医疗救治体系项目建设。广泛开展全民健身运动，提高竞技体育水平。努力创建国家卫生城市。加强国防教育，搞好国防动员、人民防空和民兵预备役建设，进一步做好创建全国“双拥”模范城工作。继续做好民族宗教、盐业管理、审计、统计、史志、对台、侨务、档案、气象、残疾人、老龄等工作。

（十）加强社会主义精神文明建设，推进民主法制进程

深入进行十六大精神和“三个代表”重要思想的宣传教育。大力加强社会主义道德建设，进一步提高公民素质和城乡文明程度。以道德规范进万家活动为载体，把群众性精神文明创建活动引向深入。进一步做好创建文明城市、文明单位、文明行业、文明景区、文明社区、文明村镇工作，促进精神文明建设发展。

坚持依法行政。《行政许可法》今年7月1日将正式施行，各级政府和政府各部门必须认真执行。要按照集中办理、统一办理、联合办理的原则，相对集中行政许可权，进一步办好市、县两级行政审批服务中心。加强法制宣传教育，深入推进“四五”普法工作。强化行政执法监督，促进严格执法、公正执法和文明执法。实行执法责任制和执法过错追究制，完善并严格执行行政赔偿制度，做到有权必有责、用权受监督、侵权要赔偿，全面提高依法行政水平。

自觉接受人大及其常委会的监督，依法向人大及其常委会报告工作，认真落实有关决议、决定。加强与政协的沟通和协商，把政治协商纳入政府决策程序，为民主监督和参政议政创造良好条件。认真办理人大代表议案、建议和政协委员提案。主动加强与各民主党派、工商联、人民团体、无党派人士的联系，充分发挥工会、共青团、妇联等组织的桥梁纽带作用。健全基层群众自治组织和民主管理制度，保证人民群众依法行使民主权利

强化社会治安综合治理，继续深入开展“严打”整治斗争，下大力气整治治安乱点，严厉打击各类刑事犯罪、经济犯罪和邪教组织的渗透破坏活动。进一步建立和完善以110为龙头的快速反应机制，搞好基层安全创建活动，努力构建治安长效机制。高度重视群众信访工作，妥善处理人民内部矛盾；认真开展排查工作，努力把矛盾解决在基层，尽可能减少群体性上访和越级上访

加大整顿和规范市场经济秩序力度。重点抓好直接关系人民群众身体健康和生命安全的食品药品等方面的专项整治，严厉打击制售假冒伪劣产品、医疗器械、农资等违法犯罪行为。继续开展整治企业经营环境专项活动，坚决制止“四乱”行为。对破坏经济秩序、严重影响企业经营环境、损害平顶山形象的重大案件，要依法从严从快查处。

四、大兴求真务实之风，树立政府良好形

新一届政府肩负着全面建设小康社会的历史使命，必须大力弘扬求真务实精神，大兴求真务实之风，树立科学的发展观和正确的政绩观，坚持以人为本，执政为民，大力推进服务型政府建设，塑造求实、创新、廉洁、高效的全新政府形象。

树立科学的发展观，切实解决发展什么、怎么发展的问题。我市正处于资源开发的高峰期，资源型城市特有的结构环境等问题不容忽视，实施二次创业，推进经济转型，必须坚持以人为本，推进经济社会的全面、协调、可持续发展，既要注重经济建设，又要注重社会进步；既要注重城市建设，又要注重农村发展；既要注重改善硬环境，又要注重优化软环境；既要注重近期发展，更要注重长远发展。坚持一切从实际出发，按科学规律办事。要在转变增长方式上下工夫，不能只讲投入不讲产出，要在加快发展的同时，保证资源的充分合理利用及环境的不断改善，做到为官一任，造福一方，惠及后代，决不能吃祖宗饭，断子孙路。

树立正确的政绩观，切实解决创什么样的政绩、怎样创政绩的问题。我们的权力是人民给的，执政的目的是为人民服务。政绩如何，不是自己说了算，而要看群众是否满意。要把为最大多数人谋利益作为一切工作的出发点和落脚点，作为判断是非的根本原则和最高标准；切实把农民增收、职工增资、财政增长、企业增效作为衡量干部政绩的基本内容；真正把群众的呼声作为“第一信号”，把群众的满意作为“第一追求”。制订政策，要充分了解民情，看看是否符合群众的意愿；落实政策，要充分依靠群众，求得大多数人的支持；评价政绩，要充分听取群众意见，看看大多数人是否满意。政绩是干出来的，不干半点政绩也没有。在其位，就必须谋其政，要把解放思想与实事求是更好地统一起来，悉心谋事，躬身实践，少说多做，真抓实干。要把对上级负责与对人民群众负责真正一致起来，把更多的时间和精力用在帮助基层和群众解决实际问题上，把更多的财力和物力用在经济社会协调发展和提高人民生活水平上，多做打基础、管长远的工作，多做让人民群众普遍受惠、长期受益的事情，使一切工作都能经得起实践、历史和群众的检验。

弘扬求真务实精神，必须继续转变政府职能。按照社会主义市场经济的要求，切实把政府经济管理的职能转变到主要为市场主体服务和创造良好发展环境上来，把政府社会管理的职能转变到提供良好的公共服务和更多的公共产品上来。实现从审批型政府向服务型政府、从全能型政府向责任型政府、从封闭型政府向开放型政府、从高成本政府向高效率政府的根本转变，使政府责任与公民权力双重回归。要选准突破口，强力推进政府效能建设。一是政府向人代会报告的工作要事事追踪，件件落实；二是政府发文要坚持少而精，做到令行禁止，确保贯彻到位；三是政府及组成部门向社会的承诺要说到做到，有诺必践。所有重大决策，都要在深入调查研究、广泛听取意见、进行充分论证的基础上，由集体讨论决定。要进一步建立健全社情民意反映制度、政府重大事项公示制度和社会听证制度，完善决策目标、执行责任、考核监督体系，充分发挥法律监督、群众监督、舆论监督的综合效能。

弘扬求真务实精神，必须切实转变干部作风。各级领导干部要带头大力弘扬焦裕禄精神、红旗渠精神和愚公移山精神，真正扑下身子、放下架子，深入基层、深入群众，掌握真实情况，解决实际问题，力求把涉及人民群众利益的每一项工作做好。要在抓落实上下苦工夫，在求实效上下真工夫，做任何工作既要雷厉风行，敢抓敢管，又要兢兢业业，扎扎实实。上面布置的工作，一定要拿出切实可行的办法，实实在在地向前推进。各级政府和政府各部门要树立全局观念，自觉地把本地区、本部门的工作融入到全市工作大局中去，对市委、市政府的决策和工作部署要不折不扣地贯彻落实，使每一项工作都能抓到细处、落到实处。坚决反对形式主义、官僚主义，力戒虚名，注重实际；力戒浮躁，注重实践；力戒空谈，注重实干；力戒形式，注重实效。要发扬“两个务必”的优良传统，牢固树立公仆意识，时刻保持清醒头脑，自重、自省、自警、自励，自觉做到廉洁自律，严格遵守反腐倡廉的各项规定，自觉接受党和人民的监督，经受住权力和金钱的考验。

各位代表，平顶山是一座充满生机与希望的城市，过去，40多年，平顶山人曾经创造出令人瞩目的成就，全面建设小康社会，为我们展现了光辉灿烂的美好前景。我们要倍加珍惜历史机遇，倍加珍惜当前大好局面，在市委的正确领导下，抢抓机遇，埋头苦干，为实施二次创业，打造经济强市，率先实现全面建设小康社会的宏伟目标而努力奋斗！
